# Cystic Periventricular Leukomalacia Worsens Developmental Outcomes of Very-Low-Birth Weight Infants with Intraventricular Hemorrhage—A Nationwide Cohort Study

**DOI:** 10.3390/jcm11195886

**Published:** 2022-10-05

**Authors:** Jong Ho Cha, Nayeon Choi, Jiyeong Kim, Hyun Ju Lee, Jae Yoon Na, Hyun-Kyung Park

**Affiliations:** 1 Department of Pediatrics, Hanyang University Hospital, Hanyang University College of Medicine, Seoul 04763, Korea; 2 Biostatistical Consulting and Research Lab, Medical Research Collaborating Center, Hanyang University, Seoul 04763, Korea; 3 Clinical Research Institute of Developmental Medicine, Hanyang University Hospital, Seoul 04763, Korea

**Keywords:** cystic periventricular leukomalacia, intraventricular hemorrhage, preterm infants, nationwide cohort, neurodevelopmental impairment, cerebral palsy, cranial ultrasonography

## Abstract

Cystic periventricular leukomalacia (cPVL) is a major brain injury involving periventricular white matter that leads to neurodevelopmental impairment in very-low-birth weight (VLBW) infants. We investigated the neurodevelopmental outcomes (motor, cognition, visual, and hearing) of 5734 VLBW infants born between 2013 and 2019 and enrolled in the Korean Neonatal Network. Cranial ultrasound results were stratified by the presence of cPVL and severity of intraventricular hemorrhage (IVH) (no, low-grade [I/II], high-grade [III]). Neurodevelopmental impairment was evaluated using cerebral palsy for motor and Bayley Scales of Infant Development for cognition. cPVL was associated with motor, cognitive, and visual impairments in those without IVH and with low-grade IVH in pairwise comparisons (Cochran–Mantel–Haenszel *p* < 0.001). Conversely, cPVL was non-significantly correlated with cognitive impairment in high-grade IVH. In regression models adjusted for neonatal variables, isolated cPVL was strongly associated with motor (22.04; 11.39–42.63) and cognitive (3.10; 1.54–6.22) impairments. This study underlines the overall considerable significance of cPVL on NDI with divergent impacts depending on the severity of IVH and developmental indices.

## 1. Introduction

Cystic periventricular leukomalacia (cPVL) is a major brain injury involving periventricular white matter that leads to neurodevelopmental impairment (NDI) in preterm infants, especially those under 32 weeks of gestational age (GA). It starts with coagulation necrosis of cellular materials mainly in the watershed zone, with impaired perfusion resulting from hypoxic-ischemic injuries [1]. Neuronal cells in the affected white matter area are depleted with severe necrosis and end up with cystic lesions [2]. While its incidence ranges from 2–7% in most countries and has been decreasing, its impact on NDI still remains significant [3,4]. Intraventricular hemorrhage (IVH) is another type of major brain injury that originates from damage to the germinal matrix vasculature, resulting in the disturbance of cerebral blood flow [5]. Similar to their different pathophysiologies, cPVL and IVH have different risk factors, according to previous studies [6,7].

cPVL and IVH have long been recognized as the major brain abnormalities responsible for NDI in very-low-birth weight (VLBW) infants (i.e., preterm infants born with birth weights of less than 1500 g). Wang et al. [7] and O’Shea et al. [8] found that preterm infants with both cPVL and IVH had worse neurodevelopmental outcomes than those with isolated cPVL. However, there was potential for confounding effects, as those studies did not control for the grade or severity of IVH. Previous literatures regarding cranial ultrasound abnormalities with NDI investigated either one of them [9,10,11,12]. Considering that approximately half of VLBW infants with cPVL also have IVH, confounding effects should be investigated [3].

Herein, by analyzing the nationwide registry data of VLBW infants, the study’s objective is to investigate the effect of cPVL on NDI using pairwise comparisons stratified by IVH.

## 2. Materials and Methods

### 2.1. Study Population

**Figure 1:**
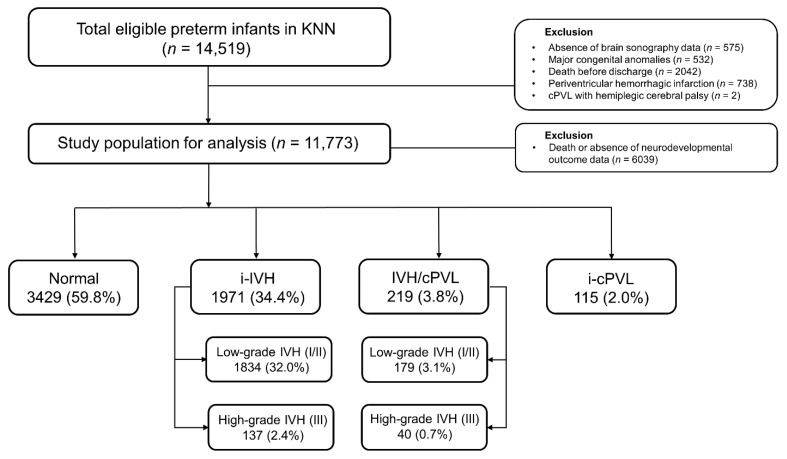
Flow chart of the study population. Abbreviations: KNN, Korean Neonatal Network; cPVL, cystic periventricular leukomalacia; IVH, intraventricular hemorrhage; i-IVH, isolated IVH; IVH/cPVL, IVH with cPVL; i-cPVL, isolated cPVL.

The Korean neonatal network (KNN) is a nationwide prospective registry of VLBW infants born in 70 tertiary hospitals in South Korea, covering more than 70% of VLBW infants. Preterm infants with birth weights of 1500 g or less, born or transferred within 28 days after birth to the neonatal intensive care units (NICUs) of the KNN, were included in the registry. Written informed consent was obtained at each NICU participating in the KNN before enrollment, and all methods were approved by the institutional review board (IRB No. 2013-06-025-043). The included population was VLBW infants in the KNN registry born between 2013 and 2019. There were 14,519 VLBW infants registered in the KNN registry, and infants with major congenital anomalies (*n* = 575), infants without cranial ultrasound findings (*n* = 532), and infants who died before NICU discharge (*n* = 2042) were excluded (Figure 1). Periventricular hemorrhagic infarction (PVHI), which is referred to as IVH grade IV, originates from impaired drainage of the medullary veins, rather than as an extension or severe form of IVH. Since we intended to investigate the effect of cPVL on the outcome of VLBW infants with IVH, infants diagnosed with PVHI (*n* = 738) were excluded from our study. In cranial ultrasonography, cystic lesions of white matter caused by cPVL could be confounded with PVHI [13]. We excluded infants diagnosed with cPVL who had hemiplegia (*n* = 2) to quantify misdiagnosis.

For the primary analysis, we sub-grouped enrolled VLBW infants into four groups: (1) normal or infants without abnormalities in cranial ultrasonography, (2) infants with isolated IVH (i-IVH), (3) infants with isolated cPVL (i-cPVL), and (4) infants with both IVH and cPVL (IVH/cPVL). In subsequent neurodevelopmental outcome analysis, we further controlled the severity of IVH (none, grade I-II as low, grade III as high) to investigate the impact of cPVL on NDI. In other words, three pairwise comparisons were implemented as follows: (1) VLBW infants without IVH (cPVL vs. without cPVL), (2) VLBW infants with low-grade IVH (cPVL vs. without cPVL), and (3) VLBW infants with high-grade IVH (cPVL vs. without PVL).

### 2.2. Cranial Ultrasonography

cPVL and IVH were diagnosed by cranial ultrasonography at each NICU hospital. Cranial ultrasonography was performed twice during the first 7 days, weekly during the 2–4 weeks after birth, and monthly until discharge. Ultrasonography was performed by well-trained pediatric radiologists or pediatricians. cPVL was defined as cystic lesions on the periventricular area. IVH was defined as hyperechoic lesions in caudothalamic or intraventricular lesions and graded as I (hemorrhage confined to the germinal matrix), II (hemorrhage without ventricular dilatation), and III (hemorrhage with acute ventricular dilatation) [14]. The worst grade among all cranial ultrasonography examinations during NICU admission was used in the analysis. PVHI was diagnosed if hemorrhagic lesions in white matter were evident in the serial cranial ultrasonography, even if evident IVH was not observed in the intraventricular area.

### 2.3. Neonatal Variables

Antenatal factors consisted of maternal factors, including maternal age, multiple births, gestational diabetes mellitus (GDM), pregnancy-induced hypertension (PIH), acute chorioamnionitis, preterm premature rupture of membrane (PPROM), completion of antenatal steroids, and type of delivery. Completion of antenatal steroids was defined as when the full injection schedule (e.g., completion of a single course of betamethasone (every 24 h for two doses) or dexamethasone (every 12 h for four doses) was accomplished. Perinatal factors consisted of GA, birth weight, head circumference at birth, Apgar score, resuscitation at birth, and blood gas analysis at birth. Neonatal factors consisted of morbidities in the NICU, including respiratory distress syndrome (RDS), mechanical ventilation for more than 7 days, systemic steroid usage, bronchopulmonary dysplasia (BPD) worse than moderate, neonatal seizure, culture-proven sepsis, necrotizing enterocolitis (NEC, ≥ stage 2 according to the modified Bell’s stage), systemic hypotension, grade III or higher retinopathy of prematurity (ROP), and duration of NICU admission. The diagnosis of BPD was confirmed based on the need for supplementary oxygen at 28 days of age, and its severity was assessed at 36 weeks of postmenstrual age. BPD worse than moderate included VLBW infants diagnosed with moderate (need for < 30% oxygen at 36 weeks of post menstrual age) or severe (≥ 30% oxygen or positive pressure) BPD [15].

### 2.4. Neurodevelopmental Outcome Analysis

At 18 to 24 months of corrected age (CA), neurodevelopmental outcomes were assessed with the use of a diagnosis of cerebral palsy (CP) and Bayley Scales of Infant Development (BSID) 2nd or 3rd edition, depending on each center’s routine protocol. VLBW infants with moderate to severe motor impairment were defined as VLBW infants diagnosed with CP, with a gross motor function classification system level of at least II to V [16]. Classification of CP included diplegia, hemiplegia, tetraplegia, and others (without mentioning detailed type). Neurological examination and diagnosis of CP were performed by experts in pediatric neurology or pediatric rehabilitation.

The BSID test evaluated the cognitive development of children aged 12–42 months, with an average of 100 and a standard deviation of 15 [17]. VLBW infants with moderate to severe cognitive impairment were defined as follows: (1) VLBW infants with a Mental Development Index (MDI) score on the BSID-II of less than 70 and (2) VLBW infants with a cognitive composite score on the BSID-III of less than 85. A previous study showed a more than 97% agreement rate between an MDI score of 70 on the BSID-II and a cognitive composite score of 85 on the BSID-III as cut-off values [18]. As the evaluation of NDI in VLBW infants includes BSID-II and BSID-III, removing the psychomotor index composite score of the BSID-II and motor composite score of the BSID-III in the evaluation of NDI is currently recommended by the National Institute of Child Health and Human Development Neonatal Research Network (USA). Similar cut-off values were introduced by Younge et al. [19]. Diagnosis of cognitive impairment with interpretation of the BSID test were confirmed by experts in pediatric neurology and by pediatric neonatologists.

We included visual and hearing impairments in those of 18 to 24 months CA as a neurodevelopmental outcome. Visual impairment was defined as ongoing treatment or follow-up with an ophthalmologist for an ophthalmologic disorder originating from prematurity. Disorders such as blindness, visual impairment, strabismus, refractive error, glaucoma, cataract, or retinopathy of prematurity (ROP) were included. Hearing problems were defined as ongoing treatment or follow-up with an otolaryngologist for deafness or hearing impairment. The definition of NDI used in the study is summarized in Table 1.

### 2.5. Statistical Analysis

Differences in baseline demographic and clinical factors among VLBW infants (normal vs. i-IVH vs. i-cPVL vs. IVH/cPVL) were compared by the Chi-square test for categorical variables and the Kruskal–Wallis test for continuous variables. In NDI analysis, the Cochran–Mantel–Haenszel (CMH) test was used to analyze the association between cPVL and NDI stratified by IVH. Then, the Chi-square test and Fisher’s exact test were performed as appropriate in pairwise comparisons. Bonferroni correction was used to reduce errors due to multiple comparisons, and the significance level was set to 0.003 (5%/15). To examine the association of cranial ultrasound abnormalities with NDI (motor and cognitive impairment) after adjustment for widely known variables, we performed a generalized linear model analysis. Results were presented with an adjusted odds ratio (OR) and 95% confidence interval (C.I). Adjusted variables included sex, GA, birth weight, PPROM, acute chorioamnionitis, postnatal steroid usage, prolonged mechanical ventilator usage, Apgar score at 5 min < 5, BPD moderate and severe, culture proven sepsis, neonatal seizure, and NEC grade ≥ II. Statistical analysis was performed using SAS version 9.4 (SAS Institute Inc., Cary, NC, USA).

## 3. Results

### 3.1. Study Participants

Of the 14,519 VLBW infants enrolled in the KNN during the study period, 11,773 were included in the initial analysis. Supplementary Table S1 summarizes the clinical characteristics between infants with and without cPVL. The cPVL group had significantly lower GA and lower birth weight. Moreover, they had a significantly higher proportion of neonatal morbidities, including BPD, NEC, and frequent postnatal steroid use.

**Figure 2:**
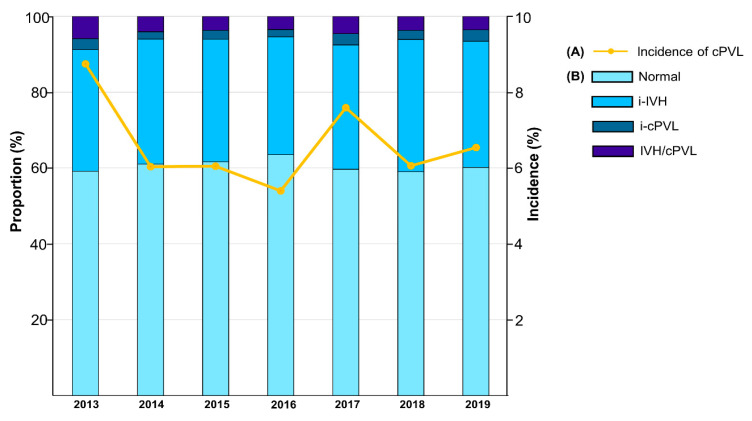
(**A**) The proportions of cranial ultrasonography abnormalities by birth year. (**B**) Incidence of cPVL by birth year. Abbreviations: cPVL, cystic periventricular leukomalacia; IVH, intraventricular hemorrhage; i-IVH, isolated IVH; IVH/cPVL, IVH with cPVL; i-cPVL, isolated cPVL.

The incidence of cPVL was 6.5%. The annual incidence of cPVL decreased from 8.8% in 2013 to 5.4% in 2016, and its incidence increased from 2017 through 2019 (6.5% in 2019). The annual incidence of cranial ultrasound abnormalities is shown in Figure 2. The incidence of ultrasound abnormality was stationary, ranging from 37–41%. The incidence of i-IVH and IVH/cPVL was also stationary, ranging from 31–34% and 3–5%, respectively.

Clinical characteristics of the participating VLBW infants are presented in Table 2. Most of the variables showed significance among sub-groups (*p* < 0.001), except for maternal age, GDM, and antenatal steroids. Compared to the rest of groups, the infants with IVH/cPVL group had the highest proportion of low GA, perinatal distress, and low birth weight. They were also likely to have neonatal morbidities, including RDS, BPD moderate to severe, seizure, sepsis, and NEC ≥ grade II (*p* < 0.001 in all variables).

For NDI analysis, 5734 VLBW infants were evaluated between 18 to 24 months of CA. In detail, 2558 VLBW infants underwent cognitive evaluation, including 1433 infants with BSID-II (56.0%) and 1125 infants with BSID-III (44.0%).

### 3.2. Pairwise Comparisons of Neurodevelopmental Outcome (cPVL vs. Non-cPVL)

The neurodevelopmental outcome of VLBW infants was classified based on the diagnosis of cranial ultrasonography: normal, cPVL, low-grade IVH, and high-grade IVH (Table 3). The incidence of motor (26.2%) and cognitive impairment (36.3%) was the highest in the cPVL group, followed by the high-grade and low-grade IVH groups. Among the cPVL group with CP, the most common type was diplegia, followed by quadriplegia. Among infants with IVH, both groups had a comparable incidence of diplegia and quadriplegia. Six infants with low-grade IVH were diagnosed with hemiplegic CP.

The NDI results in the six sub-groups based on cranial ultrasonography are shown in Table 4. After adjustment for IVH, cPVL was associated with motor, cognitive, and visual impairments (CMH *p* < 0.001). cPVL was significantly associated with moderate to severe motor, cognitive, and visual impairments in VLBW infants in groups without IVH and low-grade IVH (*p* < 0.001). Specifically, cognitive outcome was significantly worsened when cPVL was diagnosed, both in infants without IVH (13.8% vs. 30.6%, *p* < 0.001) and with low-grade IVH (20.9% vs. 37.6%, *p* < 0.001), respectively. On the other hand, the cognitive outcome of the high-grade IVH group was not significantly different according to cPVL. Finally, cPVL was not significantly associated with hearing impairment (CMH *p* = 0.329).

### 3.3. Association between Cranial Ultrasound Abnormatlities and NDI

Table 5 exhibits the results of the generalized linear models. In the crude model, compared to normal group, the IVH/cPVL group had the highest OR, followed by i-cPVL and i-IVH. After adjustment, the i-cPVL group was observed to have the highest association (adjusted OR; 22.04 [11.39–42.63] for motor, 3.10 [1.54–6.22] for cognition), followed by IVH/cPVL (adjusted OR; 16.26 [9.45–27.98] for motor, 2.29 [1.41–3.72] for cognition). For i-IVH, only motor function was significantly associated (adjusted OR; 1.76 [1.08-2.84]).

## 4. Discussion

This nationwide prospective cohort study demonstrated that cPVL was associated with NDI in VLBW infants without IVH and with low-grade IVH. The incidence of cPVL in the study was 6.5%, which is consistent with a previous report (3–10%) [20]. Unlike previous studies that demonstrated a decreasing incidence of cPVL, the incidence of both cPVL and IVH did not improve in our study [21,22].

The IVH/cPVL group had significantly higher rates of neonatal morbidities. Differences in such events (e.g., RDS, BPD, sepsis, NEC, ROP) imply that the IVH/cPVL group experienced more frequent systemic hypoxia/ischemia and inflammatory events in the NICU environment than the i-IVH group. In the neurodevelopmental outcome analysis, we studied NDI in VLBW infants with cPVL. To our knowledge, this is the first study to investigate the effect of cPVL on NDI stratified by IVH, using nationwide population-based cohort data. In the CMH test for general association, cPVL was associated with moderate to severe motor impairment after adjustment for IVH. This finding is consistent with previous studies that showed that cPVL was a strong predictor of motor impairment [12,23]. In the pairwise comparisons, cPVL was associated with worsened motor function in VLBW infants in every grade of IVH. This supports previous studies that revealed cPVL affects periventricular structures (e.g., corticospinal tract), followed by subsequent cortical volume reduction and functional connectivity impairments [24,25]. In our study, cPVL worsened the neurodevelopmental outcome in VLBW infants with low-grade IVH. Given that low-grade IVH is an independent risk factor for NDI in VLBW infants [11,26], the risk increases when cPVL is combined. cPVL is known to affect the preterm brain holistically, not only the white matter. Infants with cPVL are at risk for grey matter injury, as demonstrated by magnetic resonance imaging (MRI) studies showing a reduction of cortical volume, as well as microstructural alterations [27,28]. The link between IVH and cPVL could be explained by the loss of oligodendrocyte progenitor cells (OPCs), which in turn, develop into pre-myelinating oligodendrocytes and finally mature into myelinating oligodendrocytes. IVH damages OPCs by releasing blood products, and the damages would result in reduced myelination, with white matter damage [5].

On the other hand, in infants with high-grade IVH, the effect of cPVL was significant in only in motor functions. We speculate that VLBW infants with high-grade IVH may have white matter damage that is not reliably detected in conventional ultrasonography. Back et al. [2] proposed that mild hypoxic-ischemic injury, which is not sufficient to cause necrotic change of white matter, could lead to selective degeneration of pre-oligodendrocytes. Moreover, Wagenaar et al. [29] reported an MRI study showing that IVH was a significant factor in punctate white matter lesions defined as focal patches located in periventricular white matter. The risk factors for these white matter lesions were IVH, surfactant, and postnatal intubation. Thus, we assume that clinicians may consider the presence of high-grade IVH as a sign of latent white matter damage, even though cPVL has not been witnessed. Since brain MRI examination during NICU admission is challenging, follow-up brain MRI imaging after discharge is essential. In the analysis of high-grade IVH, we excluded PVHI cases, since both cPVL and PVHI could evolve into cystic lesions of the white matter in the preterm brain [13]. Compared to PVHI, ultrasonographic finding of cPVL have a tendency of bilateral, symmetric echodensities in the dorsolateral region of lateral ventricles. Relative sparing of the more laterally positioned pyramidal tracts would result in diplegic CP, whereas PVHI cases would result in more hemiplegic CP [30]. We excluded cases of PVHI and cPVL with hemiplegic CP to quantify the genuine impact of cPVL.

In cognitive function, the association of cPVL was significant in the pairwise comparisons in VLBW infants without IVH and low-grade IVH. Given that preterm infants achieve language and cognitive milestones as they age, the effect of cPVL on cognitive impairment is likely to increase with age. This possibility is supported by previous studies showing associations between cPVL and learning disabilities in preschool-aged children [31,32,33]. We assume that contrasting results between motor and cognitive function in high-grade IVH group originated from the limitation of evaluating whole cognitive functions in 18–24 months of CA. Therefore, a long-term prospective study is needed to elucidate the prolonged effect of cPVL on cognitive function. The overall incidence of visual and hearing impairments were higher than a previous study [3]. In particular, cPVL was associated with visual dysfunction in VLBW infants without IVH and low-grade IVH [34]. This suggests that direct white matter injury caused by cPVL is a critical risk factor for visual dysfunction, compared with indirect white matter damage caused by low-grade IVH [35,36]. Our study highlights that there were some subtle distinctions depending on the development index and the severity of IVH.

Interpretation of other risk factors that could hamper NDI is noted when analyzing the impact of brain abnormalities. As the known risk factors, such as BPD and postnatal steroid usage, were present in different proportions among sub-groups, additional analysis controlling for those variables were performed, which revealed contrasting results between crude and adjusted models. However, caution is required in interpretation that i-cPVL should not be regarded as more dangerous abnormality than IVH/cPVL. Rather, it indicates that white matter damage per se could cause lethal impact on NDI [8].

The strengths of the study lie in a relatively large cohort of VLBW infants. This is also the first nationwide prospective cohort study in South Korea on neurodevelopmental outcome stratified by the results of cranial ultrasonography. However, the current study has several limitations. First, the rates of long-term follow-up were relatively low. There is a risk of underestimating the neurodevelopmental outcome. However, the rate of cPVL between the follow-up group and without follow-up group was similar (5.8% vs. 7.1%). In KNN protocol, participants performed one of three developmental assessments: BSID test, Korean Ages & Stages Questionnaires test (K-ASQ), and Korean developmental screening test (K-DST) at 18–24 months of CA. All 70 centers of KNN perform developmental assessments with their protocol. We only included infants who performed the BSID test in cognitive function analysis, since both K-ASQ and K-DST are developmental screening tests with parent-answered questionnaires. Thus, the number of infants who had their cognitive functions evaluated was lower than those with motor, visual, and hearing functions. Second, we defined NDI by different cut-off values for BSID-II and BSID-III. The cut-off value of BSID-III was higher, based on previous studies showing that BSID-III can overestimate an infant’s developmental performance [18,37]. Lastly, since the KNN database does not provide ultrasound images, we could not assess the detailed information of lesions (e.g., location, size) that could affect the neurodevelopmental outcome and evaluate inter and intra-observer reliability. In addition, as the clinical sensitivity of neuro-ultrasound is challenging, it does not ascertain that the latent abnormalities of the normal group. Nonetheless, ultrasound is the most easily accessible diagnostic tool in the NICU environment and its clinical significance should be noted.

## 5. Conclusions

This prospective nationwide cohort study showed that after adjustment for IVH, cPVL was associated with NDI in VLBW infants. In detail, cPVL was a worsening factor for motor impairments in all IVH groups and cognitive impairments in without IVH and low-grade IVH groups. We emphasize the importance of radiologic workup for the early detection of cPVL in preterm infants with IVH, allowing early intervention to improve neurodevelopmental outcomes.

## Figures and Tables

**Table 1:** Definition of neurodevelopmental impairment used in the study.

Neurodevelopmental Impairment	Definition
Motor impairment	Normal to mild motor impairment: (1) VLBW infants without cerebral palsy (2) VLBW infants diagnosed with cerebral palsy with GMFCS level I
	Moderate to severe motor impairment: (1) VLBW infants diagnosed with cerebral palsy with GMFCS level II or greater (II–V)
Cognitive impairment	Normal to mild cognitive impairment: (1) VLBW infants with a BSID-II MDI score ≥70 (BSID-II) (2) VLBW infants with a BSID-III cognitive composite score ≥ 85 (BSID-III)
	Moderate to severe cognitive impairment: (1) VLBW infants with BSID-II MDI score < 70 (BSID-II) (2) VLBW infants with BSID-III cognitive composite score < 85 (BSID-III)
Visual impairment	Blindness, Strabismus, Refractive error, Glaucoma, Cataract, Retinopathy of prematurity
Hearing impairment	Deafness, Hearing loss

Abbreviations: VLBW, very low-birth weight; GMFCS, gross motor function classification system; BSID, Bayley Scales of Infant and Toddler Development; MDI, Mental Development Index.

**Table 2:** Clinical characteristics of the participating VLBW infants, according to the results of cranial ultrasonography.

Variables	Normal (*n* = 7150)	i-IVH (*n* = 3858)	i-cPVL (*n* = 288)	IVH/cPVL (*n* = 477)	*p* Value
Maternal age, years	33.0 (31.0–36.0)	33.0 (31.0–36.0)	33.0 (31.0–36.0)	33.0 (31.0–36.0)	0.92
Sex (Male)	3463 (48.4)	1898 (49.2)	140 (48.6)	271 (56.9)	<0.001
Multiple births	2755 (38.5)	1305 (33.8)	109 (37.8)	176 (36.9)	<0.001
Gestational diabetes	707 (9.9)	403 (10.4)	31 (10.8)	55 (11.5)	0.57
PIH	1771 (24.8)	869 (22.5)	46 (16.0)	72 (15.1)	<0.001
Acute chorioamnionitis	1833 (30.1)	1229 (37.3)	66 (28.6)	141 (35.3)	<0.001
PPROM	2310 (32.5)	1383 (36.1)	117 (41.1)	193 (40.7)	<0.001
Completion of antenatal steroid	3310 (57.5)	1828 (57.1)	120 (53.3)	209 (52.8)	0.27
Caesarean section	5874 (82.2)	3017 (78.2)	236 (81.9)	364 (76.3)	<0.001
Gestational age (weeks)					<0.001
GA < 28	1507 (21.1)	1690 (43.8)	85 (29.5)	244 (51.2)	
28 ≤ GA < 32	4015 (56.2)	1751 (45.4)	181 (62.8)	216 (45.3)	
GA ≥ 32	1628 (22.8)	417 (10.8)	22 (7.6)	17 (3.6)	
Apgar at 5 min < 5	333 (4.7)	426 (11.1)	27 (9.4)	77 (16.2)	<0.001
Resuscitation at birth	6026 (84.7)	3527 (92.1)	267 (92.7)	459 (96.2)	<0.001
pH at birth < 7	46 (0.8)	74 (2.5)	4 (1.9)	6 (1.8)	<0.001
BE at birth < −12	176 (3.1)	168 (5.7)	9 (4.2)	19 (5.8)	<0.001
Birth weight (g)					<0.001
Birth weight < 1000	1626 (22.7)	1649 (42.7)	82 (28.5)	219 (45.9)	
1000 ≤ Birth weight < 1250	2157 (30.2)	1053 (27.3)	84 (29.2)	127 (26.6)	
Birth weight ≥ 1250	3367 (47.1)	1156 (30.0)	122 (42.4)	131 (27.5)	
RDS	4858 (67.9)	3190 (82.7)	250 (86.8)	438 (91.8)	<0.001
Prolonged mechanical ventilation, ≥7 days	1665 (23.3)	1895 (49.1)	116 (40.3)	311 (65.2)	<0.001
Postnatal steroid use	1031 (14.4)	1368 (35.5)	80 (27.8)	238 (49.9)	<0.001
BPD moderate and severe	1405 (19.9)	1596 (41.7)	80 (27.8)	274 (57.4)	<0.001
Neonatal seizure	95 (1.3)	260 (6.7)	15 (5.2)	92 (19.3)	<0.001
Culture-proven sepsis	938 (13.1)	927 (24.0)	68 (23.6)	156 (32.7)	<0.001
NEC grade ≥ II	230 (3.2)	250 (6.5)	22 (7.6)	50 (10.5)	<0.001
Systemic hypotension	817 (11.4)	966 (25.0)	61 (21.2)	195 (40.9)	<0.001
ROP grade ≥ III	447 (6.3)	666 (17.3)	21 (7.3)	96 (20.1)	<0.001

Data are expressed as median (Q1–Q3) in continuous variables and number (%) in categorical variables. Abbreviations: VLBW, very-low-birth weight; cPVL, cystic periventricular leukomalacia; IVH, intraventricular hemorrhage; i-IVH, isolated IVH; i-cPVL, isolated cPVL; IVH/cPVL, IVH with cPVL; PIH, pregnancy-induced hypertension; PPROM, preterm premature rupture of the membranes; GA, gestational age; BE, base excess; RDS, respiratory distress syndrome; BPD, bronchopulmonary dysplasia; NEC, necrotizing enterocolitis; ROP, retinopathy of prematurity.

**Table 3:** Incidence of neurodevelopmental impairment at 18 to 24 months of corrected age, according to cranial ultrasound findings.

	cPVL	Low-Grade IVH (IVH I/II)	High-Grade IVH (IVH III)
Moderate to severe motor impairments *	82 (26.2)	93 (4.8)	34 (20.2)
Diplegia	40 (12.8)	43 (2.2)	17 (10.1)
Hemiplegia	0 (0)	6 (0.3)	0 (0)
Quadriplegia	33 (10.5)	36 (1.8)	13 (7.7)
Others	9 (2.9)	8 (0.4)	4 (2.4)
Moderate to severe cognitive impairments ^†^	58 (36.3)	223 (22.6)	30 (35.3)

Data are expressed as the numbers (%). Abbreviations: VLBW, very low-birth weight; cPVL, cystic periventricular leukomalacia; IVH, intraventricular hemorrhage. * Numbers of data for whom information was obtainable; 313 with cPVL, 1957 with low-grade IVH, 168 with high-grade IVH, 5594 with overall. **^†^** Numbers of data for whom information was obtainable; 160 with cPVL, 988 with low-grade IVH, 85 with high-grade IVH, 2558 with overall.

**Table 4:** Neurodevelopmental outcomes of VLBW infant sub-groups based on the results of cranial ultrasonography.

		IVH (−)		IVH (I/II)		IVH (III)		CMH *p* Value
		cPVL (−)	cPVL (+)	cPVL (−)	cPVL (+)	cPVL (−)	cPVL (+)	
Motor impairment	Normal to mild	3331 (99.0)	84 (79.2)	1740 (97.5)	124 (72.9)	111 (84.7)	23 (62.2)	<0.001
	Moderate to severe	34 (1.0)	22 (20.8)	45 (2.5)	46 (27.1)	20 (15.3)	14 (37.8)	
	*p* value ^†^	<0.001 *^,A^		<0.001 *^,B^		0.003 *^,B^		
Cognitive impairment	Normal to mild	1238 (86.2)	34 (69.4)	707 (79.1)	58 (62.4)	46 (67.6)	9 (52.9)	<0.001
	Moderate to severe	199 (13.8)	15 (30.6)	187 (20.9)	35 (37.6)	22 (32.4)	8 (47.1)	
	*p* value ^†^	0.001 *^,B^		<0.001 *^,B^		0.256 ^B^		
Visual impairment	No	3084 (91.5)	86 (79.6)	1541 (85.2)	120 (68.2)	81 (59.6)	23 (59.0)	<0.001
	Yes	286 (8.5)	22 (20.4)	267 (14.8)	56 (31.8)	55 (40.4)	16 (41.0)	
	*p* value ^†^	<0.001 *^,B^		<0.001 *^,B^		0.948 ^B^		
Hearing impairment	No	3279 (98.8)	105 (97.2)	1731 (97.7)	159 (96.4)	126 (95.5)	37 (100.0)	0.329
	Yes	39 (1.2)	3 (2.8)	41 (2.3)	6 (3.6)	6 (4.5)	0 (0.0)	
	*p* value ^†^	0.145 ^A^		0.285 ^A^		0.341 ^B^		

Data are expressed as the numbers (%). Abbreviations: VLBW, very-low-birth weight; IVH, intraventricular hemorrhage; cPVL, cystic periventricular leukomalacia; CMH, Cochran–Mantel–Haenszel. ^†^ Pairwise comparison between VLBW infants (cPVL vs. non-cPVL) The significance level was corrected from 0.05 to 0.003 (5%/15) by the Bonferroni correction method. ^A^ Fisher’s exact test. ^B^ Chi-square test. * Significant after Bonferroni correction.

**Table 5:** Multivariable logistic regression model presenting the association between cranial ultrasound abnormalities and neurodevelopmental disorders.

	Crude Model		Adjusted Model ^a^	
	Odds Ratio	95% C.I.	Odds Ratio	95% C.I
Moderate to severe motor impairments				
Normal	reference		reference	
i-IVH	3.44	2.26–5.23	1.76	1.08–2.84
i-cPVL	25.66	14.39–45.75	22.04	11.39–42.63
IVH/cPVL	39.99	25.45–62.84	16.26	9.45–27.98
Moderate to severe cognitive impairments				
Normal	reference		reference	
i-IVH	1.73	1.39–2.14	1.07	0.83–1.39
i-cPVL	2.74	1.47–5.13	3.10	1.54–6.22
IVH/cPVL	3.99	2.65–6.02	2.29	1.41–3.72

Abbreviations: C.I., confidence interval; cPVL, cystic periventricular leukomalacia; IVH, intraventricular hemorrhage; i-IVH, isolated IVH; i-cPVL, isolated cPVL; IVH/cPVL, IVH with cPVL; PIH, pregnancy-induced hypertension; PPROM, preterm premature rupture of the membranes; GA, gestational age; BPD, bronchopulmonary dysplasia; NEC, necrotizing enterocolitis. ^a^ Adjusted for sex, GA, birth weight, PPROM, acute chorioamnionitis, postnatal steroid usage, prolonged mechanical ventilator usage, Apgar score at 5 min < 5, BPD moderate and severe, culture proven sepsis, neonatal seizure, and NEC grade ≥ 2.

## Data Availability

The Korean Neonatal Network (KNN) Publication Ethics Policy adheres to the following research data management and access guidelines: All information about patients and participating NICUs is confidential and is only available to individuals who have access for the purposes of the research activities permitted. Access is only allowed for the purpose of collecting data for the first time, and no access for any other purpose is allowed.

## Supplementary Materials

The following supporting information can be downloaded at: https://www.mdpi.com/article/10.3390/jcm11195886/s1, Table S1; Baseline demographic characteristics of VLBW infants in the study.
