# Supplementary material for: Cystic Periventricular Leukomalacia Worsens Developmental Outcomes of Very-Low-Birth Weight Infants with Intraventricular Hemorrhage—A Nationwide Cohort Study
Source: J Clin Med. 2022 Oct 5;11(19):5886. doi: 10.3390/jcm11195886 (PMC9572154; doi:10.3390/jcm11195886)
Supplement: Supplementary file 1 [file jcm-11-05886-s001.zip › jcm-1861309 -SUPPLEMENTARY MATERIAL.pdf]

Journal of
Clinical Medicine

MDPI

Supplementary Material

Cystic Periventricular Leukomalacia Worsens Developmental Outcomes of Very-Low-Birth Weight Infants with Intraventricular Hemorrhage—A Nationwide Cohort Study

Jong Ho Cha [1], Nayeon Choi [2], Jiyeong Kim [2], Hyun Ju Lee [1,3], Jae Yoon Na [1,*,†] and Hyun-Kyung Park [1,3,*,†]

1 Department of Pediatrics, Hanyang University Hospital, Hanyang University College of Medicine, Seoul 04763, Korea
2 Biostatistical Consulting and Research Lab, Medical Research Collaborating Center, Hanyang University, Seoul 04763, Korea
3 Clinical Research Institute of Developmental Medicine, Hanyang University Hospital, Seoul 04763, Korea
* Correspondence: hypedheart@hanyang.ac.kr (J.Y.N.); neopark@hanyang.ac.kr (H.-K.P.); Tel.: +82-2-2290-8397 (H.-K.P.); Fax: +82-2-2297-2380 (H.-K.P.)
† These authors contributed equally to this work.

Supplementary Table S1. Baseline demographic characteristics of VLBW infants in the study.

Variables	VLBW infants without cPVL (n = 11,008)	VLBW infants with cPVL (n = 765)	p value
Maternal age, years	33.0 (31.0,36.0)	33.0 (31.0,36.0)	0.59
Sex (male)	5361 (48.7)	419 (54.8)	< 0.001
Multiple births	4060 (36.9)	285 (37.3)	0.87
Gestational diabetes	1110 (10.1)	86 (11.2)	0.33
PIH	2640 (24.0)	118 (15.4)	< 0.001
Acute chorioamnionitis	3062 (32.7)	207 (32.9)	0.96
**PPROM**	3693 (33.8)	310 (40.8)	< 0.001
Completion of antenatal steroid	5138 (57.0)	329 (53.0)	0.06
Caesarean section	8891 (80.8)	600 (78.4)	0.13
Gestational age (weeks)			
GA < 28	3197 (29.0)	329 (43.0)	< 0.001
28 ≤ GA < 32	5766 (52.4)	397 (51.9)	
GA ≥ 32	2045 (18.6)	39 (5.1)	
Apgar at 5 min < 5	759 (6.9)	104 (13.7)	< 0.001
Resuscitation at birth	9553 (86.8)	726 (94.9)	< 0.001
pH at birth < 7	120 (1.4)	10 (1.8)	0.50
BE at birth < -12	344 (4.0)	28 (5.2)	0.22
Birth weight (g)			< 0.001
Birth weight < 1000	3275 (29.8)	301 (39.3)	
1000 ≤ Birth weight < 1250	3210 (29.2)	211 (27.6)	
Birth weight ≥ 1250	4523 (41.1)	253 (33.1)	
Incremental birth head circumference, cm	26.5 (25.0, 28.0)	26.0 (24.0, 27.5)	< 0.001
RDS	8048 (73.1)	688 (89.9)	< 0.001
Prolonged mechanical ventilation, ≥7 days	3560 (32.3)	427 (55.8)	< 0.001
Systemic steroid	2399 (21.8)	318 (41.6)	< 0.001
BPD moderate and severe	3001 (27.3)	354 (46.3)	< 0.001
Neonatal seizure	355 (3.2)	107 (14.0)	< 0.001
Culture proven sepsis	1865 (16.9)	224 (29.3)	< 0.001
NEC grade ≥ 2	480 (4.4)	72 (9.4)	< 0.001
Systemic hypotension	1783 (16.2)	256 (33.5)	< 0.001

ROP grade ≥ 3	1113 (10.1)	117 (15.3)	< 0.001
**Accompanied cerebral lesions**			< 0.001
Without IVH	7150 (65.0)	288 (37.6)	
IVH grade I	2809 (25.5)	229 (29.9)	
IVH grade II	782 (7.1)	150 (19.6)	
IVH grade III	267 (2.4)	98 (12.8)	

Data are expressed as the numbers (%) for categorical variables and mean (interquartile range) for continuous variables.

Abbreviations: VLBW, very-low-birth-weight; cPVL, cystic periventricular leukomalacia; PIH, pregnancy-induced hypertension; PPROM, preterm premature rupture of the membranes; GA, gestational age; BE, base excess; RDS, respiratory distress syndrome; BPD, bronchopulmonary dysplasia; NEC, necrotizing enterocolitis; ROP, retinopathy of prematurity; IVH, intraventricu.
